# Socio-affective and cognitive predictors of social adaptation in vulnerable contexts

**DOI:** 10.1371/journal.pone.0218236

**Published:** 2019-06-14

**Authors:** Alejandra Neely-Prado, Gorka Navarrete, David Huepe

**Affiliations:** Center of Social and Cognitive Neuroscience, Universidad Adolfo Ibáñez, Santiago, Chile; Iwate Medical University, JAPAN

## Abstract

People living in vulnerable environments face a harder set of challenges adapting to their context. Nevertheless, an important number of them adapt successfully. However, which cognitive and socio-affective variables are specifically related to these variations in social adaptation in vulnerable contexts has not been fully understood nor directly addressed. Here we evaluated socio-affective variables (anxious attachment style, internal locus of control, self-esteem and stress) and cognitive variables (fluid intelligence, crystallized intelligence, working memory, numeracy, probabilistic reasoning and logical reasoning) to explain variations in social adaptation in a sample of 232 adults living in vulnerable contexts (*M* = 42.3, *SD* = 14.9, equal amount of men and women). Our results show that an important amount of variance in social adaptation can be explained by socio-affective variables, principally by self-esteem, while cognitive variables also contributed significantly. As far as we know, this is one of the first steps towards understanding the role of cognitive and socio-affective features on social adaptation. In the long run, this area of research could play an important role on the assignation of resources to ease people’s integration into society. Our data and R analysis scripts can be found at: https://osf.io/egxy5/.

## Introduction

Social adaptation has been defined as the capacity to compromise, relate, confront and cooperate with the environment and others, accommodating our mental and behavioral processes [1]. Although defined, this concept has not been frequently addressed as a phenomenon by itself (e.g. [2–4]). Most studies about social adaptation do not use social adaptation scales that measure this ability directly. Instead, they commonly analyze correlations between variables that might be relevant for social adaptation, such as having a job, intellectual functioning, health-related quality of life, among others [2–4]. Although some authors show how social adaptation is negatively affected in psychological and psychiatric disorders [5, 6] and in people who had to overcome problems on initial stages of development [7–10], to the best of our knowledge there is no research on the social adaptation of healthy people living in vulnerable contexts.

To directly assess social adaptation, we used the Social Adaptation Self-Evaluation Scale (SASS) [11]. This scale measures people’s motivation and the implications of their behavior on engaging in social activities, asking about hobbies, family life, work, relationships, intellectual interests, environment managing capacity and perception of self-performance.

Literature has shown evidence that growing up in poverty, predicts difficulties in several dimensions such as emotion and behavior regulation, and academic failure [12–14]. Although vulnerability and poverty are not the same, they generally coexist. Poverty is typically defined by a specific SES (Socio Economic Status) threshold [15]. Vulnerability is a more complex construct, that includes a SES threshold, and also other aspects as living in environments characterized by limited access to social security, education, housing, jobs and health. Since 2015, the Chilean Social Development department defines poverty in this multidimensional way that equals the vulnerability concept described above [16]. We will analyze which socio-affective and cognitive variables predict better levels of social adaptation among vulnerable contexts. A secondary goal will be to assess if socio-affective or cognitive features are more relevant for this process.

It seems important to acknowledge that even though we are studying social adaptation among adults, early experiences are in many occasions crucial to the development of socio-affective and cognitive variables [17–19]. For example, it has been seen that early experiences with a mother suffering from depression can predict the apparition of eating disorders by the time children meet adolescence [20]. Thus, many of the variables that we included and which we expect to behave as predictors of social adaptation have been seen to develop during life’s early stages [10, 14, 21, 22]. Keeping the above in mind, we made an exhaustive literature search that allowed us to identify several variables that could be significant predictors of social adaptation. We also saw that these variables could be grouped in socio-affective and cognitive features. In the following sections, we will review each variable expected to predict social adaptation in vulnerable contexts in this study, and the justifications for including them in a hierarchical multiple regression analysis. To do so, we will review first the socio-affective variables, and then the cognitive variables.

### Socio-affective variables

A persons’ social environment, and social and affective characteristics, are important predictors of several psychological and coping difficulties. More specifically, it has been suggested that socio-affective features give rise to coping strategies that lead to better adjustment and well-being [23–26]. Also, when difficulties are seen in socio-affective dimensions of the individual, proneness to psychopathology and psychiatric symptoms tend to arise [27–30]. On these studies, self-esteem, stress and attachment are repeatedly mentioned as important aspects that determine vulnerability vs well-adjustment when facing traumatic or anxious situations and life events. Although locus of control is less mentioned, there is an extended amount of literature that suggests it might be of some importance to social adaptation (for references and more details see below in the section “Locus of Control”).

Thus, although there could be several socio-affective variables to consider, we will consider the following ones suggested by the literature, and discuss their possible relationship to social adaptation on the next lines: anxious attachment, locus of control, stress and self-esteem. Although our aim is not to understand the specific mechanisms between socio-affective variables, we would like to clarify the importance they may have on predicting social adaptation in vulnerable contexts, and then offer some underlying mechanisms between the proposed socio-affective and cognitive models.

It has been suggested that the relational and cognitive tendencies that shape our representations of the world are strongly influenced by attachment [31]. And because early social deprivation has been shown to predict higher levels of insecure attachment [8], we believe that insecure attachment, specifically the anxious type, will be over represented in people living in vulnerable contexts and impact negatively in their capacity to adapt. Insecure attachment styles (anxious and avoidant) are associated with difficulties in several functions [25, 32–34]. A study revealed that people with a avoidant attachment style, have shown to use curiosity to connect with others and the environment, and although they fear to hurt others feelings, they actively try to gain as much information as possible, which reduces probabilities of isolation [25]. On the other hand and according to the same study, people with anxious attachment style, tend to use curiosity as a way to gain control over their relationships as the same study describes. The latter are now perceived as threats which can lead to isolation. Thus, it has been argued that people with an anxious attachment style have more difficulties than people with avoidant attachment when managing emotions during decision-making or interpreting reality [35–40]. In this sense, it is possible that an anxious attachment style could lead to less social adaptation abilities in vulnerable contexts.

Locus of control, meanwhile, refers to the tendency of people to interpret life situations as depending on its own behavior instead of as a result of luck, fate, or the influence of other powerful people or forces [41]. People with this kind of bias may engage differently in tasks and everyday decisions, affecting their capacity to adapt to social situations. Actually, it has been argued that the style of locus of control that is adopted during childhood seems related to educational attainment and health levels during the next stages of development [22]. In a study with recovering cardiac patients spouses, those who had a perception of control (associated to internal locus of control) over the disease, had a better emotional adjustment, less anxiety levels, less hostility and less levels of depression [42]. Also, it has been documented that people with higher levels of self-efficacy living in vulnerable contexts are more prone to use social support systems to improve their social adaptation [43]. Following the lead of these studies we hypothesize that an internal locus of control will be related to higher levels of social adaptation in vulnerable contexts.

Other variable that becomes of interest for the aim of this study is stress. People who live in vulnerable contexts often suffer from constant exposure to high levels of stress, which are thought to be related to social adaptation difficulties [12, 44]. With stress, we refer to the perception each person has of its environment and the amount of threat this implies for the organism’s homeostasis [45]. Stress has its roots in neurobiological mechanisms that regulate how we perceive and respond to our environment and has repercussions on the expression of aggressive and antisocial behavior [46, 47]. Some negative consequences related to high levels of stress have been seen in people with depression, making scholar achievements and functionality more difficult [48]. How the organism copes with stress has consequences in neural circuits, changing the balance between anxiety, memory, mood and decision making [49]. We suggest that high levels of perceived stress could be related to social adaptation difficulties in vulnerable contexts. In the same vein, there is evidence that chronic stress, as occurs in contexts of poverty, leads to reductions in performance in cognitive and interpersonal domains [50, 51].

Finally, when considering individual factors that can affect social adaptation from a socio-affective perspective, self-esteem also seems to play an important role. There has been an increased interest in the relationship between self-esteem and subjective well-being [52], and also its relationship with high anxiety traits that have been shown to lead to chronic health issues [53]. At the same time, social stress has shown to be related to worse self-esteem [54]. Although studies directly relating self-esteem to social adaptation are still missing, high self-esteem has been associated with higher success and well-being in interpersonal, work and health dimensions [55]. Also, low self-esteem has been linked to higher tendencies to depression or anxiety [56]. Thus, it is likely that higher self-esteem would be related to better social adaptation since it seems to have a positive impact on several psychological outcomes.

### Cognitive variables

Although there seems to be more interest and evidence on the effects of socio-affective features on coping and well-being (constructs that indirectly address social adaptation characteristics) [57, 58], some studies suggest that cognitive factors seem to support these processes [59], while cognitive factors also appear negatively affected by traumatic and stressful situations and by difficulties on emotion-regulation [60–62]. Specifically these studies suggest that aspects like working memory, long-term memory, cognitive control, and others have a role on emotion-regulation that could help coping and resilience mechanisms, while at the same time these individual characteristics face difficulties when living in adverse environments or passing through stressful situations.

As the case of socio-affective variables, there could be several cognitive variables to consider. However, we will take into account ones that may have an association to social adaption according to the literature: working memory, fluid intelligence, crystallized intelligence, numeracy, probabilistic reasoning, and logical reasoning.

Working memory is a widely studied cognitive function [63] that we suggest could help to predict social adaptation in vulerable contexts. There is some evidence that working memory is diminished in people who live in poverty [50], and it seems to be highly relevant for social adaptation because of its connections with cognitive control, behavioral inhibition, intelligence and executive functions [64–68]. All variables that have been related to important aspects of social adaptation, as academic success or self-regulation (e.g. [69–71]) Although little seems to be known about how working memory affects social adaptation, it has been found that working memory and emotional recognition are negatively related with the ability of people with schizophrenia to solve social problems [72, 73]. Also, it is known that working memory is associated to metacognitive skills [74] while these have proven to be crucial to different aspects of social adaptation on everyday life and in contexts of chronic social stress [75–78].

Strongly associated with the above, are fluid intelligence and crystallized intelligence [79, 80]. It has been suggested that these variables could be significant variables that influence social adaptation positively as well, according to an important body of previous findings. Both seem to be tied to educational attainment [81, 82], which in the case of the population included in this research, is generally low. Fluid intelligence is a cognitive capacity that has shown to be very important for several functions such as emotional regulation and theory of mind [21, 83]. Low fluid intelligence has been associated with tendencies to commit physical violence, to be a victim of physical violence, to drug consumption, to worst perception of mental health and to lower self-esteem [84]. In contrast, crystallized intelligence, or vocabulary tests used to measure crystallized intelligence, have been shown to be negatively related with behavioral impulsiveness in adolescents and academic failure [85]. Besides, crystallized intelligence seems to be necessary for concrete figurative reasoning [86], which could impact context interpretations. Although to the best of our knowledge crystallized intelligence has not shown to be directly associated with social adaptation, fluid intelligence has [84]. But because both have to do with how much information we store and our ability to use it in novel and every day situations [87], we believe that both could contribute to social adaptation.

Living in vulnerable contexts implies low access to education, which makes it difficult to achieve numerical or other types of knowledge [88–90]. In this sense, numeracy could also have an important role in predicting social adaptation in vulnerable contexts. Specifically, it has been postulated that numeracy could be an important predictor of inequalities regarding health and income because of its central role for decision making [91]. It has been documented that numeracy is negatively associated with people’s proneness to influence their decisions and judgments by emotional events that are irrelevant [92]. Numeracy seems to help people to achieve a better balance between available information and emotions, which also improves probabilistic reasoning in risky situations [93].

Interestingly, probabilistic reasoning is important when understanding our surroundings, enabling us to use novel information to update our knowledge, but also, when solving everyday life problems [94]. Although studies linking probabilistic reasoning and social adaptation are lacking, there is evidence that it affects the way people make judgments and thus, it is not surprising to find it diminished in people with psychiatric disorders [95, 96]. Besides, there is evidence indicating that social learning processes could be a product of this kind of reasoning because of its relevance for decision-making, the evaluation of environmental cues [97] and for thinking about and evaluating the future [98]. We believe that this could transfer to a positive influence of probabilistic reasoning on social adaptation.

Finally logical reasoning, as the capacity to arrive at valid conclusions from given premises [99], may also be related to people’s social adaptation. Reasoning requires filtering emotional information to distinguish relevant from irrelevant information [100] which is also useful in routine decision making. It has been shown that logical reasoning is related to cognitive ability [101], and there is evidence suggesting that cognitive ability predicts better performance in logical reasoning even when conflicting information is presented [102]. As we know, information as presented in daily tasks is not always clear and formally presented, people regularly need to correctly select and process information to make the best decisions according to their goals. We believe logical reasoning should be important for social adaptation because of its impact on information processing and decision making.

### Relationship between socio-affective and cognitive variables

Although there is evidence supporting a relationship between the above-mentioned variables and social adaptation, there is another distinction to be made: which group of variables—cognitive or socio-affective—will have greater predictive power on social adaptation.

How cognitive and socio-affective abilities differentiate and connect is still a hotly contested topic [103], and it is difficult to estimate if socio-affective or cognitive variables are more important for social adaptation, since both dimensions are deeply intertwined [104–106]. However, ‘high order cognitive functions’ (as the cognitive variables included in this study [107]) have shown to be connected with emotion regulation: emotion regulation affects cognition efficiency and vice-versa [108]. One of the aims of this research was to test the influence of both kind of variables on social adaptation in a hierarchical approach. This type of analysis was chosen to find differences in the weights of each group of variables over social adaptation.

It has been suggested that cognitive control capacity depends on emotions [109] and that emotional tendencies are important for behavior regulation [110]. Thus, it is not surprising to find increasing evidence of cognition and emotion being interdependent, because they share functions across several networks in the brain across domains [104, 111, 112]. Following this reasoning, we will analyze the influence that cognitive variables add to the relationship between socio-affective features and social adaptation. We expected to find (1) that every cognitive and socio-affective variable selected through our careful literature review (see Socio-affective variables and Cognitive variables sections for an exhaustive list) will individually show a significant correlation with social adaptation, (2) that all the selected socio-affective and cognitive variables will significantly predict social adaptation when included in a global model, (3) that the socio-affective model would predict a larger percent of social adaptation variance than the cognitive model and (4) that the cognitive model variables will improve social adaptation prediction beyond the socio-affective variables.

## Materials and methods

### Ethics statement

Every procedure of this research was approved by Universidad Diego Portales’s ethics committee. Each participant signed an informed consent which contained its rights and research details.

### Participants

A group of 232 people aged between 18 and 89 (*M* = 42.3, *SD* = 14.9, equal amount of men and women), with 94.7% of the sample between 18 and 65 years old (excluding the older participants from the analysis made no qualitative difference) were recruited by accessibility.

Participants were adults from the communes of La Granja and San Joaquín (Santiago, Chile) who belong to the 40th percentile of the Chilean welfare program (Programa Social Chile Solidario del Ministerio de Desarrollo Social). The Chilean welfare program grants subsidies, bonuses and other facilities to people who are living in a vulnerable situation. For people or families to receive these benefits, they must be registered to a social index card that indicates the percentile of vulnerability of the person or family. To calculate these percentile, their socioeconomic status and the access they have to public services are taken into account. People or families in the 40th percentile are considered as living in a vulnerable situation and adults from this group were included in this study.

Educational level of participants was ranged between “Primary school, not completed” (1) and “Bachelor degree completed or more” (8), but the maximum educational level achieved by people from this sample was of 5, where 1 = “Primary school, not completed” (22,8%), 2 = “Primary school, completed” (15,1%), 3 = “High school, not completed” (30,6%), 4 = “High school completed”(23,7%), 5 = “Technical degree not completed”(7,8%), 6 = “Technical degree completed”, 7 = “Bachelor degree not completed”, 8 = “Bachelor degree completed or more”. Plus, two other categories: 9 = “No studies”, 10 = “I don’t know/ it doesn’t apply”. People were interviewed to be certain they didn’t have any psychiatric or psychological disorder, and then they proceeded to the evaluation session, where they completed a set of computerized tests and questionnaires. This was done in one session of approximately 2 hours duration. Data collection was part of two FONDECYT research projects (N° 1171200, of PI David Huepe and N° 1171035 of PI Gorka Navarrete).

### Materials

Participants were requested to answer several tests and questionnaires. We selected a group of constructs that, according to the revised literature, could help to answer our main question about the relevance of cognitive and socio-affective features on social adaptation. Each construct was measured by a single test or questionnaire. Demographic information such as age, education and socioeconomic level (by income) was also collected. We used Cronbach’s Alpha to determine the reliability of the scales.

#### Social adaptation

This construct was measured using the Social Adaptation Self evaluation Scale (SASS) [11], a 21 item questionnaire that assesses various aspects of social life, like the capacity of maintaining a job, interpersonal relationships, hobbies, etc. Each item has 4 answering options that are scored from 0 to 3. All items need to be answered, except for items 1 and 2, where only one of them must be answered depending if the person has a job or not. Answering options vary for each item (see example below). The scale has a maximum score of 60 points, where the higher the score the more socially adapted a person is considered to be. We used the Spanish validation of this instrument [113], obtaining an optimal level of reliability (*α* = 0.8). See for example item 7:

“Is the state of relations with your family:”

0) unsatisfactory, 1) fair, 2) good, 3) very good

#### Attachment

We used the Chilean validation of the ECR-RS test to measure participants’ attachment styles [114, 115]. It is a 36 (9 x 4) item questionnaire used to classify people’s attachment style (secure, insecure avoidant or insecure anxious attachment). The same 9 items are used in 4 sections, to assess attachment to the mother, the father, the person’s romantic partner and friends, respectively. Each item has 3 response options: 0 meaning “no”, 1 meaning “yes”, and 2 meaning “does not apply”. Our literature review showed that only anxious attachment styles could be a good predictor of social adaptation, so for this study, we only took into account the anxious dimension of the scale. Specifically, this consists in the mean of responses on items 7, 8 and 9 across the 4 sections. The higher the score, the more anxiously attached each person was considered to be. The mean per subject was calculated summing the responses for all items (0 or 1 in 12 items; 3 items x 4 sections) and then dividing it by the number of items (12). There were no “2” answers, so no special treatment was needed for calculating total scores. In our sample, test reliability was of *α* = 0.87 for the anxiety dimension.

7) “I usually worry that I don’t longer matter to this person”
8) “I fear this person can abandon me.”
9) “I worry that this person doesn’t care for me as much as I care for him or her.”

0) No, 1) Yes, 2) Does not apply

#### Self-esteem

To measure self-esteem, we selected the Rosenberg’s scale [116]. This scale consists in 10 items answered in a Likert scale ranging from 1 (“Totally Disagree”) to 4 (“Totally Agree”). In this case a Chilean validation was used [117]. Scores vary between 10 and 40, higher scores meaning higher self-esteem. Items 3, 5, 8, 9, 10 were reversed (*α* = 0.81). For example, item 9:

“Sometimes I feel that I am truly useless”

1 Totally Disagree—4 Totally Agree

#### Locus of control

As our literature review showed the importance of Internal Locus of control for several variables related to social adaptation, we used the “Internality” dimension of the Chilean validated version of the Levenson questionnaire [118, 119]. This dimension consists of eight items (items 1, 4, 5, 9, 18, 19, 21 and 23). We summed the items from this dimension to achieve an “internality measure” as suggested in the literature [119]. Answers range from 1 (“Totally Agree”) to 6 (“Totally disagree”), so the higher the score in the “Internality” dimension, the more internal the person’s attribution style is considered to be. The test showed an adequate reliability for the “Internality” dimension (*α* = 0.71). For example, Item 1:

“That I become a leader depends mostly of my own abilities.”

1 Totally Agree—6 Totally disagree

#### Stress

To address stress we used the “Stress” dimension of the General Health Questionnaire (GHQ-12) [120]. The GHQ-12 is a 12 item questionnaire aimed at measuring three different dimensions: “Adaptive success” (items 1, 3, 4, 7, 8 and 12), “Self-Esteem” (items 6, 9, 10 and 11), and “Stress” (items 2, 5 and 9). A Spanish validated adaptation was used [120], with answers ranging from 0 to 3 according to the frequency each situation is experienced by the person. Higher the scores on the “Stress” dimension, are considered to reflect higher levels of perceived stress. The “Stress” dimension of the GHQ-12 questionnaire had an adequate reliability (*α* = 0.74). For example, item 5:

“Felt constantly under strain”

0) No, not at all, 1)Not more than usual, 2)More than usual, 3)Much more than usual

#### Fluid intelligence

We selected the matrices sub-test of the WAIS-III [121] test to measure fluid intelligence. For all WAIS-III tasks we used the Spanish version [121]. In this test people have to choose which of the given alternatives completes a pattern shown. For correct answers, one point is given, for incorrect answers, no point is given. There are 26 trials, so 26 is the maximum direct score. We used the standardized scores, which range between 1 and 18. The test showed high reliability for the sample of this study (*α* = 0.92). For instance, in front of a sequence pattern like:

○ o ○ o ○ ____

What is the correct alternative? 1) ○, 2) ∪, 3) Δ, 4) o, 5) □

#### Crystallized intelligence

We used the vocabulary sub-test of the WAIS-III [121] test to measure crystallized intelligence. This subtest consists in 33 words which meaning has to be explained (e.g. item 19 “evolve”). Each answer is classified according to its similarity to the correct definition, specified in the WAIS-III manual. Answers get 0 points when the definition is considered null, 1 point if it is partially correct and 2 points when it is complete. The subtest has a direct score up to 66 points. We used the standardized scores, which range from 1 to 19 points. The higher the score, the higher the persons crystallized intelligence is considered to be (*α* = 0.84).

#### Working memory

We selected the digit sub-test of the WAIS-III [121] test to measure working memory. This subtest consists in 9 series of digits that the person has to repeat in the reversed order (e.g. in item 2: “5-8-2”, people should respond “2-8-5”). The test has 7 span levels (progressively increasing the number of digits), with two trials each. The first span level starts with three-digit sequences, if the person gives a correct answer on the first trial, the next span level is shown, if the person gives an incorrect answer on the first span level, the second trial in the same span level is shown. If the person gives a correct answer in the second trial then it proceeds to the next span level, but if it gives an incorrect answer on the second trial the test finishes in that span level. The person has to give incorrect answers on both trials of one span level to suspend the test. The span level in which the test gets suspended corresponds to the final score.

#### Numeracy

To evaluate levels of numeracy we used the Lipkus Numeracy Scale [122]. This test has a total of 11 items where people have to solve simple arithmetic problems, and where the person gets one point for each correct answer. Our research group translated the original instrument through a double-blind method, and it showed adequate reliability levels in this study (*α* = 0.71). For example, item 1:

“Imagine that we roll a fair, six-sided dice 1,000 times. Out of 1,000 rolls, how many times do you think the dice would come up even: 2, 4, or 6?: ____”

#### Probabilistic reasoning

To measure probabilistic reasoning, specific problem solving tasks were designed following other research’s approaches [123–126] in which people had to solve probabilistic problems and then make decisions from numerical estimates. The following is an example of a question of this test:

10 out of 1000 50 year old women that take part in a massive screening test have breast cancer

9 of those 10 women with cancer will receive a positive mammogram

20 out of 990 women that don’t have cancer will also receive a positive mammogram

Of the women that receive a positive mammogram, how many would you expect to have cancer?

Answer: __ out of __

#### Logical reasoning

We assessed logical reasoning using 23 syllogisms [100, 127–129]. In a logical syllogism, two premises and a conclusion are given. The premises are assumed to be true, and the participant has to answer if the conclusion logically follows the premises. The measure of logical reasoning is the sum of accurate responses (*α* = 0.63 and *α* = 0.65 after eliminating item 3).

“Every creature that’s born from an egg are lizards.

Some birds are lizards.

Some birds are not born from an egg”.

[TRUE]

### Statistics

The group of variables used in this research often have been found to correlate among each other, and/or to social adaptation. To replicate correlations found in previous literature, we collected descriptive information (see Table 1) and then searched for bivariate correlations between variables (see Table 2).

**Table 1:** Descriptive statistics for our sample on measures of cognitive and socio-affective features and demographic variables.

	N	Mean	CI	St. Dev.	Min	Max	Range
Social Adaptation	226	40.2	(39.1,41.2)	7.9	14	57	0-60
Internal Locus	230	40.2	(39.2,41.2)	7.7	6	48	0-48
Self-Esteem	231	27.7	(27.1,28.3)	4.9	10	35	10-40
Anxious Attachment	231	0.4	(0.31,0.39)	0.3	0	1	0-1
Stress	227	3.79	(3.49,4.10)	2.3	0	9	0-9
Working Memory	232	6.8	(6.45,7.11)	2.5	1	14	0-9
Fluid Intelligence	230	5.9	(5.58,6.30)	2.8	1	15	0-26
Crystallized Intelligence	231	6.6	(6.29,6.83)	2.1	1	12	0-19
Logical Reasoning	229	8.4	(8.02,8.81)	3.0	0	15	0-23
Probabilistic Reasoning	228	0.3	(0.26,0.33)	0.3	0	1	0-1
Numeracy	232	3.2	(2.94,3.53)	2.3	0	8	0-11
Age	232	42.3	(40.4,44.2)	14.9	18	89	18-89
Education Level (EL)	232	2.8	(2.62,2.95)	1.3	1	5	1-5

*Note*: N = amount of people that completed each of the tests or questionnaires (out of 232); Mean = average scores (please see above for details about each instrument’s correction); CI = confidence intervals; St. Dev. = standard deviations for each measure; Min = minimum score in the present sample; Max = maximum score in the present sample. Range = Range of possible scores for each measure. For Education Level: 1 = “Primary school, not completed”, 2 = “Primary school, completed”, 3 = “High school, not completed”, 4 = “High school completed”, 5 = “Technical degree not completed”

**Table 2:** Correlation matrix.

	Social Adaptation	Internal Locus of Control	Self-Esteem	Anxious Attachment	Stress	Working Memory	Fluid Intelligence	Crystallized Intelligence	Logical Reasoning	Probabilistic Reasoning	Numeracy	Sex	Age
Social Adaptation													
Internal Locus	0.34***												
Self-Esteem	0.47***	0.34***											
Anxious Attachment	-0.18**	-0.11	-0.22***										
Stress	-0.39***	-0.18**	-0.32***	0.15*									
Working Memory	0.22***	0.11	0.16*	0.07	-0.01								
Fluid Intelligence	0.30***	0.02	0.17*	-0.13*	-0.11	0.31***							
Crystallized Intelligence	0.27***	-0.05	0.23***	-0.27***	-0.11	0.41***	0.49***						
Logical Reasoning	0.15*	0.24***	0.14*	-0.01	-0.02	0.17*	0.27***	0.06					
Probabilistic Reasoning	0.12	0.15*	0.17*	-0.07	-0.09	0.23***	0.22***	0.05	0.30***				
Numeracy	0.23***	0.04	0.22***	-0.11	-0.11	0.18**	0.40***	0.26***	0.31***	0.27***			
Sex	0.05	0.00	0.11	0.04	-0.16*	0.05	0.10	0.17**	0.04	0.02	0.13*		
Age	0.00	-0.03	0.03	-0.18**	0.09	0.22***	0.09	0.53***	-0.19**	-0.27***	-0.27***	0.13*	
Educational Level (EL)	0.29***	0.07	0.15*	-0.01	-0.12	0.19**	0.33***	0.15*	0.32***	0.27***	0.42***	-0.02	-0.41***

*Note*: Pearson correlations *r* and levels of significance.

*p<0.1;

**p<0.05;

***p<0.01

Hierarchical multiple regression analysis was used to determine the contribution of each variable to the prediction of social adaptation, and to test if cognitive variables add a significant amount of explained variance to the prediction of socio-affective variables over social adaptation (see Table 3). Hierarchical regression analysis is a specific method for evaluating changes in predictability of one group of independent variables over and above another group of independent variables [130]. Thus, variables are entered in different steps to the regression analysis, testing if variables entered in a second step predict a significant amount of variance over and above those variables entered in a first step in the analysis. This analysis will allow us to observe if socio-affective variables, entered first in the hierarchical regression analysis, predict social adaptation and if there predictability is augmented when cognitive variables are entered in a second stage.

**Table 3:** Hierarchical regression coefficients.

	*Dependent variable*:		
	Socio-affective Model	Social Adaptation Cognitive Model	Complete Model
	(1)	(2)	(3)
Internal Locus of Control	0.161** (0.038, 0.284)		0.173*** (0.048, 0.298)
Self-Esteem	0.577*** (0.371, 0.784)		0.489*** (0.284, 0.694)
Anxious Attachment	-1.479 (-4.569, 1.610)		-0.861 (-4.019, 2.297)
Stress	-0.783*** (-1.191, -0.375)		-0.730*** (-1.125, -0.334)
Working Memory		0.363 (-0.086, 0.813)	0.347* (-0.058, 0.752)
Fluid Intelligence		0.336 (-0.118, 0.790)	0.383* (-0.008, 0.775)
Cryst. Intelligence		0.620** (0.013, 1.227)	0.335 (-0.225, 0.895)
Logical Reasoning		0.189 (-0.172, 0.550)	0.019 (-0.299, 0.338)
Prob. Reasoning		0.790 (-3.306, 4.886)	-1.334 (-4.910, 2.243)
Numeracy		0.384 (-0.120, 0.888)	0.157 (-0.283, 0.597)
Constant	21.375*** (14.240, 28.510)	28.600*** (24.251, 32.950)	15.797*** (8.220, 23.374)
Observations	213	213	213
R^2^	0.318	0.159	0.387
Adjusted R^2^	0.305	0.134	0.357
F Statistic	24.213*** (df = 4; 208)	6.470*** (df = 6; 206)	12.758*** (df = 10; 202)
ΔR^2^			0.069*** (df = 6; 202)

*Note*:

*p<0.1;

**p<0.05;

***p<0.01

*Note*: This table presents non-standardized beta values and confidence intervals for each cognitive and socio-affective variable in each model. The table also presents the results of each step of the hierarchical regression

For each step of the statistic analysis we used R [131]. Data and R analysis scripts can be found at: https://osf.io/egxy5/.

## Results

### Bivariate correlations

Self-esteem (r = .47, p<0.001), stress (r = -.39, p<0.001) and internal locus of control (r = .34, p<0.001) displayed moderate correlations with social adaptation according to Pearson’s r correlation coefficients [132]. Fluid intelligence (r = .30, p<0.001), crystallized intelligence (r = .27, p<0.001), working memory (r = .22, p<0.001), and numeracy (r = .23, p<0.001) showed moderate to weak significant correlations. Though still significant, only a weak correlation was found between anxious attachment (r = -.18, p<0.01) and logical reasoning (r = .15, p<0.05) and social adaptation. Probabilistic reasoning was not significantly related to social adaptation (r = .12, p>0.05). See Table 2 for a detailed account of correlations and p-value thresholds.

Our analysis suggests that higher self-esteem, crystallized intelligence, fluid intelligence, numeracy, working memory, less stress levels and internal locus of control, contribute to social adaptation in vulnerable contexts. In general, socio-affective variables showed a stronger relationship with social adaptation than cognitive variables.

### Hierarchical regression

For the hierarchical regression analysis we used socio-affective variables (anxious attachment, self-esteem, stress and internal locus of control) and cognitive variables (fluid intelligence, crystallized intelligence, working memory, numeracy, probabilistic reasoning and logical reasoning) as predictors of social adaptation scores. The analysis revealed that the set of socio-affective variables explain a significant proportion of the social adaptation scores’ variance, adj. R^2^ = .305, F(4,208) = 24.21, p<.001. Although the set of cognitive variables explained less than the set of socio-affective variables by itself, adj. R^2^ = .134, F(6, 206) = 6.47, p<.001, they still explained a significant proportion of the variance, when added to the socio-affective variables, ΔR^2^ = .07, F(6,202) = 3.81, p<.01.

**Fig 1:**
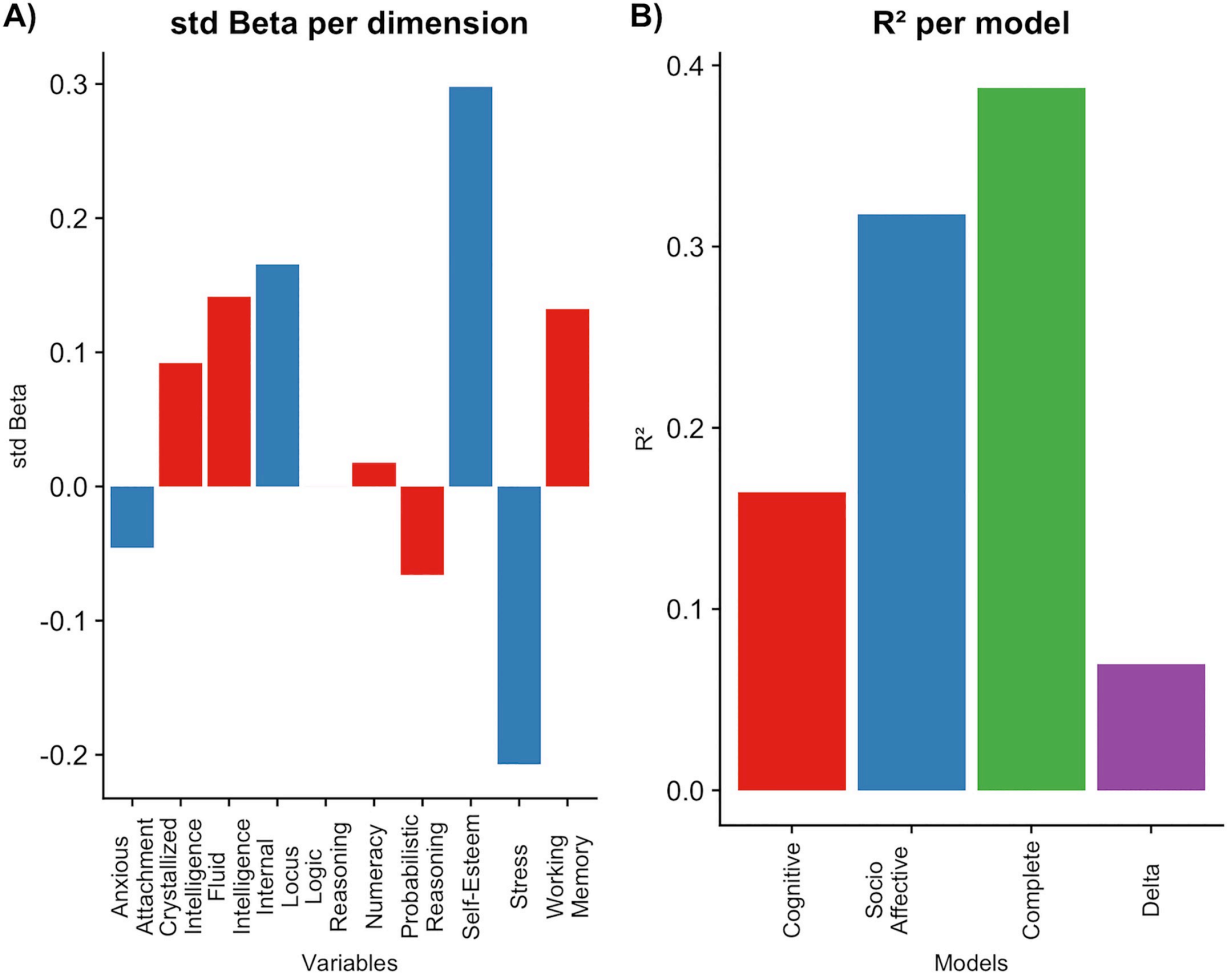
Contribution of each group to the dependent variable’s variance. A) Standardized beta values of each independent variable in predicting social adaptation B) R² values of the Cognitive and Socio-affective models; the complete model that includes the cognitive variables over the socio-affective variables; and the delta R², that shows the contribution of the cognitive model over the socio-affective model.

The complete model (socio-affective plus cognitive variables) explained an important amount of variance in social adaptation, adj. R^2^ = .357, F(10, 202) = 12.76, p<.001. Sample normality was proved using Shapiro-Wilk analysis, W = 0.994, p>.05 and variation inflation factor confirmed that the amount of explained variance was not inflated because of correlation between predictors (VIF < 1.6). The details of the hierarchical regression analysis can be seen in Table 3 and Fig 1.

Both models (socio-affective and cognitive) explain a significant amount of the variance of social adaptation, with the socio-affective variables contributing on a larger scale to this relationship. Finally, we were able to determine that cognitive variables contributed to predicting social adaptation beyond the socio-affective variables. 31,8% percent of the variance in social adaptation was accounted for by socio-affective variables. Locus of control, stress and self-esteem contributed significantly to the model, but anxious attachment did not. On the other hand, cognitive variables accounted for 7% of the variance of social adaptation after socio-affective were included in the model, but only fluid intelligence and working memory contributed significantly to this relationship. The final model, considering both sets of variables, explained a total of 38,7% of social adaptation variance.

## Discussion

Our aim was to understand which psychological aspects help social adaptation in people living in vulnerable contexts. The hypotheses tested were the following: (1) crystallized intelligence, fluid intelligence, working memory, logical reasoning and probabilistic reasoning—as cognitive variables—and self-esteem, locus of control and anxious attachment—as socio-affective variables—correlate significantly with social adaptation; (2) on one hand the socio-affective model, and on the other hand the cognitive model, predict an important amount of social adaptation variance; (3) the socio-affective model included predicts a larger percent of social adaptation variance than the cognitive model included in this study; (4) this cognitive model improves social adaptation prediction beyond that provided by the socio-affective model.

Data analysis showed that the variables that were selected through literature revision do correlate with social adaptation, except for the case of probabilistic reasoning. A floor effect on the test used to measure this variable could explain why it did not show a significant correlation. Also, self-esteem showed the strongest correlation with social adaptation, which goes in line with previous findings that establish that self-esteem relates to subjective well-being and success in different areas such as personal relationships, work and health [52, 55].

The hierarchical regression analysis revealed our model to predict a significant amount of social adaptation variance (38,7%), although socio-affective variables showed to be of greater importance and not all variables contributed to the model’s predictive capacity.

Within socio-affective variables, an unexpected outcome was that anxious attachment did not contribute to the model’s predictive power, which may be a characteristic of this particular sample. This was the only socio-affective variable that did not contribute to the model’s predictability.

The cognitive set of variables we selected also showed to be relevant to predict social adaptation, although none of these variables by itself appeared as a significant predictor when included in the complete model. Crystallized intelligence showed to be a significant predictor in the cognitive model. The high impact of SES on crystallized intelligence [133] could explain why it appeared as a relevant predictor of social adaptation in the cognitive model.

Also, it seems that the contribution of the cognitive model to social adaptation is highly distributed among its variables, which could explain why in the complete model none of the individual cognitive variables is explaining a significant amount of variance by itself. When observing the beta values of working memory, fluid intelligence and crystallized intelligence in the complete model, it’s possible to note that these variables make similar contributions to social adaptation (they also showed stronger bivariate correlations with social adaptation). However, here we show that in a vulnerable population sample, when these cognitive variables and others are integrated into a model, the shared variance between them becomes the predictor of social adaptation. Future work should consider larger samples to better understand the role, for instance, of fluid intelligence and working memory and its relationship with other cognitive and socio-affective variables.

Socio-affective variables showed greater predictive power than cognitive variables, which is consistent with what researchers have found about emotions regulating cognitive abilities [108, 109, 134]. However, the integrative model that includes both socio-affective and cognitive variables showed to predict an important percentage of social adaptation variance, which is also aligned with evidence showing that cognitive and emotional features interplay to cope with the environment [104, 111, 112].

We studied how emotion and cognition help to shape social adaptation in vulnerable populations. Even though most studies on poverty emphasize its negative effects, our findings show a healthy amount of variability in several socio-affective and cognitive traits related to social adaptation. This suggests that there are traits that help people to cope when living in a vulnerable context. High levels of self-esteem showed to be an outstanding predictor, together with stress and internal locus of control. The finding that socio-affective variables play a major role in social adaptation could be good news for policy making and educational institutions, given that these can be meaningfully improved [135–138]. This contrasts with the limited or null transference of cognitive abilities training to real-world domains [139–142].

Nonetheless, most of the cognitive measures in our sample were on the low end of the distribution, so a relevant inquiry that should be made in the future, is if the presence of higher cognitive abilities could increase their predictive power on social adaptation to levels similar of those of socio-affective variables.

Our findings seem to be aligned with the idea that emotions play an important role for the generation and promotion of cognition, and that both are interdependent [143] in responding to the environment. Predictive power shown by cognitive variables are compatible with literature suggesting that cognitive features are of great importance to different aspects of adaptation [144, 145], we can see that cognitive variables explain less variance of social adaptation once we controlled by socio-affective variables, which suggests that both sets of variables share some common features. However, further research is needed to gain better understanding on the relationship between cognition and affection, and how they can influence core capacities such as social adaptation.

## Conclusion

As far as we know, this is the first study showing that cognitive (working memory and fluid intelligence) and socio-affective (self-esteem, stress and locus of control) variables predict social adaptation among adults living in vulnerable contexts. High self-esteem showed the be the strongest predictor of social adaptation, while low levels of stress, internal locus of control and high levels of fluid intelligence and working memory were also critical. Specifically, 31,8% of differences in social adaptation were accounted by stress, internal locus of control, and self-esteem, while 7% depended on working memory and fluid intelligence. Because this line of research has not been deeply addressed, further research is still needed. Nonetheless, these findings suggest that public policies could consider self-esteem, locus of control and perceived stress as relevant areas to intervene, and that cognitive and socio-affective variables should be considered as mechanisms that work together to produce behavior.

## Limitations

Because we used self-reported questionnaires to measure socio-affective variables and social adaptation, we collected people’s self-perception. Inevitably, this could affect the outcomes with well-known bias such as desirability. Also, as people living in vulnerable environments generally have low levels of education, it’s possible some of the participants didn’t fully understand some of the items (like probabilistic reasoning that showed floor effects). Nevertheless, we systematically observed that the majority of correlations and effects were consistent with the hypothesis proposed in this study. Although, to lessen these limitations we used reliability analyses, with generally good results.

Finally, we did not have a control group to compare if our findings are specific of people living in vulnerable contexts or if they could be extrapolated to the general population. However, Latin America has a great amount of population living under this multidimensional poverty, although this situation has shown some improvement in the last decade [146, 147]. Within Latin America, Chile is a country with an important amount of vulnerable population as well [16], and for this reason, understanding social adaptation mechanisms in this group is interesting in itself. Also, it is possible to speculate that the subgroup of people with high social adaptation in our study could have similar cognitive and socio-affective characteristics than the general population’s social adaptation levels. For example, the average scores in self-esteem for the subgroup with high social adaptation, are similar to the general population. The Rosenberg’s Self-Esteem scale [117] has a mean of 32.47 in the Chilean general population, while a mean of 30.85 was obtained in a study including people from 53 nations [148]. In this study, those who obtained a “perfectly adapted” score (SASS total score > 48 points) [11], reached a mean of 30.7 in the Rosenberg’s Self-Esteem scale. Nevertheless, future research should be developed to assess this outstanding issue.
